# Predictors of right ventricular function and size in patients with hypertrophic cardiomyopathy

**DOI:** 10.1038/s41598-020-78245-x

**Published:** 2020-12-03

**Authors:** Mateusz Śpiewak, Mariusz Kłopotowski, Łukasz Mazurkiewicz, Ewa Kowalik, Joanna Petryka-Mazurkiewicz, Barbara Miłosz-Wieczorek, Anna Klisiewicz, Adam Witkowski, Magdalena Marczak

**Affiliations:** 1 Magnetic Resonance Unit, Department of Radiology, National Institute of Cardiology, ul. Alpejska 42, 04-628 Warsaw, Poland; 2 Department of Interventional Cardiology and Angiology, National Institute of Cardiology, Warsaw, Poland; 3 Department of Cardiomyopathies, National Institute of Cardiology, Warsaw, Poland; 4 Department of Congenital Heart Diseases, National Institute of Cardiology, Warsaw, Poland; 5 Department of Coronary Artery Disease and Structural Heart Diseases, National Institute of Cardiology, Warsaw, Poland

**Keywords:** Cardiology, Cardiomyopathies

## Abstract

We investigated factors associated with right ventricular (RV) function and size in hypertrophic cardiomyopathy (HCM) patients. Two hundred fifty-three consecutive HCM patients and 20 healthy volunteers underwent cardiac magnetic resonance examination. In addition to measuring RV function (ejection fraction—RVEF) and size (end-diastolic volume—RVEDV), each image was inspected for the presence of RV and left ventricular (LV) hypertrophy, and the maximal wall thickness of the left and right ventricles was recorded. HCM patients had higher RVEF and lower RVEDV than healthy volunteers and similar RV mass. The mean RV wall thickness was higher in HCM patients than in controls. LV late gadolinium enhancement (LGE) was present in 89.7% of patients, and RV LGE was present in 3.1% of patients (p < 0.0001). Univariate and multivariable analyses revealed that LVEF, peak LV outflow tract gradient, LV LGE, maximal LV wall thickness, and tricuspid regurgitation (TR) volume by magnetic resonance imaging were positive predictors of RVEF. In addition to TR volume, the only independent predictor of RVEF < 45% was LVEF (odds ratio = 0.80, 95% confidence interval 0.67–0.95). Multivariable analysis revealed that LVEDV and TR volume were positive predictors of RVEDV, whereas negative predictors were RVEF, maximal RV wall thickness, LV LGE, and age. Neither estimated systolic pulmonary artery pressure nor TR grade by echocardiography proved to be predictors of RVEF. There were no differences in either the maximal RV wall thickness or the maximal left ventricular (LV) wall thickness in patients stratified according to NYHA functional class (p = 0.93 and p = 0.15, respectively). There were no differences in mean RV wall thickness in patients categorised based on the number of clinical risk factors for sudden cardiac death (SCD), i.e., non-sustained ventricular tachycardia, family history of SCD, or unexplained syncope (p = 0.79). On the other hand, there was a weak positive association between RV hypertrophy and the estimated probability of SCD at 5 years (rho = 0.16, p = 0.01). RV systolic dysfunction measured as decreased RVEF was uncommon in HCM and was associated with poor LV systolic function. LV also had a significant impact on RV size.

## Introduction

Hypertrophic cardiomyopathy (HCM) is the most common inherited disease of the human myocardium^1^. For unknown reasons, it primarily affects the left ventricle, while in the vast majority of cases, the right ventricle is spared from hypertrophy. However, several studies employing cardiac magnetic resonance imaging (MRI) have revealed that myocardial hypertrophy also involves the right ventricle^2–6^. No studies have exclusively addressed the issue of predictors of right ventricular (RV) size and function in HCM patients. Accordingly, we aimed to assess RV size and function in a large prospectively gathered cohort of HCM patients.

## Results

The analysis included 253 HCM patients and 20 healthy volunteers. The majority of patients were asymptomatic (New York Heart Association—NYHA—functional class I) or mildly symptomatic (NYHA II) in terms of heart failure symptoms (Table 1). There were no patients in NYHA IV functional class. HCM patients were older than control subjects (Table 1). HCM patients had higher RVEF, similar RVM, and lower RVEDV than healthy volunteers (Table 1). Consequently, the mean RV wall thickness and RVM indexed for RVEDV (0.21 ± 0.05 vs. 0.17 ± 0.02 g/mL, p < 0.0001) were higher in HCM patients than in controls (p < 0.0001, Table 1). There were no differences in either the maximal RV wall thickness or the maximal left ventricular (LV) wall thickness in patients stratified according to NYHA functional class (p = 0.93 and p = 0.15, respectively). There were no differences in mean RV wall thickness in patients categorised based on the number of clinical risk factors for sudden cardiac death (SCD), i.e., non-sustained ventricular tachycardia, family history of SCD, or unexplained syncope (p = 0.79). On the other hand, there was a weak positive association between RVH and the estimated probability of SCD at 5 years (rho = 0.16, p = 0.01).

**Table 1:** Baseline characteristics of the study group.

	HCM patients (n = 253)	Control group (n = 20)	p-value
Age (years)	49.1 ± 15.2	29.0 ± 5.6	< 0.0001
Sex (males/females)	149/104 (58.9% males)	10/10 (50% males)	0.44
RVEF (%)	64.6 ± 8.3%	55.7 ± 4.5%	< 0.0001
RVM (g/m^2^)	17.5 ± 3.8	17.3 ± 2.3	0.79
RVEDV (mL/m^2^)	84.4 ± 16.6	103.3 ± 11.8 mL/m^2^	< 0.0001
Mean RV wall thickness (mm)	4.8 ± 2.4	2.3 ± 0.4	< 0.0001
LVEF (%)	64.3 ± 8.1	60.7 ± 3.0	0.0001
LVM (g/m^2^)	82.8 ± 16.1	54.2 ± 8.8	< 0.0001
LVEDV (mL/m^2^)	92.1 ± 19.1	95.0 ± 7.1 s	0.16
LV wall thickness (mm)*	20.0 (17.0–24.0)	8.5 (7.0–9.0)	< 0.0001
**NYHA functional class**		–	–
I	133 (52.6%)		
II	77 (30.4%)		
III	43 (17.0%)		
IV	0 (0%)		
NSVT on Holter monitoring	39 (15.4%)	–	–
Family history of SCD	22 (8.7%)	–	–
Unexplained syncope	27 (10.7)	–	–
Probability of SCD at 5 years* (%)	2.63 (1.84–4.0)	–	–

Unless otherwise specified, continuous data are presented as means ± SD.

*LV* left ventricular, *LVEDV* left ventricular end-diastolic volume, *LVEF* left ventricular ejection fraction, *LVM* left ventricular mass, *NSVT* non-sustained ventricular tachycardia, *RV* right ventricular, *RVEDV* right ventricular end-diastolic volume, *RVEF* right ventricular ejection fraction, *RVM* right ventricular mass, *SCD* sudden cardiac death.

*Data presented as medians with interquartile ranges in brackets.

RVM was positively and significantly correlated with LVM (r = 0.46, p < 0.0001). HCM patients had higher LVM than controls (p < 0.0001, Table 1).

**Figure 1:**
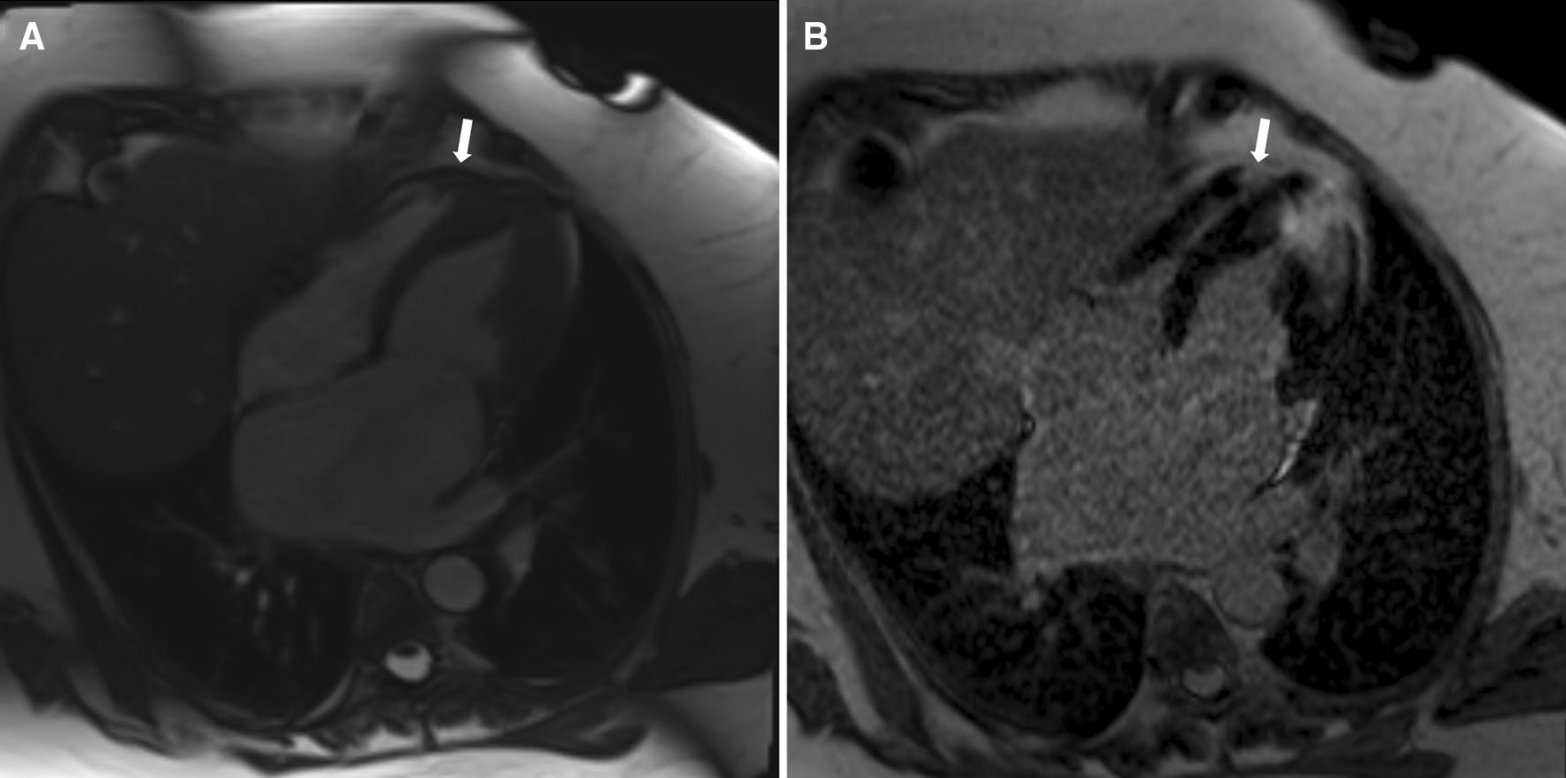
Representative images showing right ventricular hypertrophy (**A**) and late gadolinium enhancement (**B**) in the apical region.

**Figure 2:**
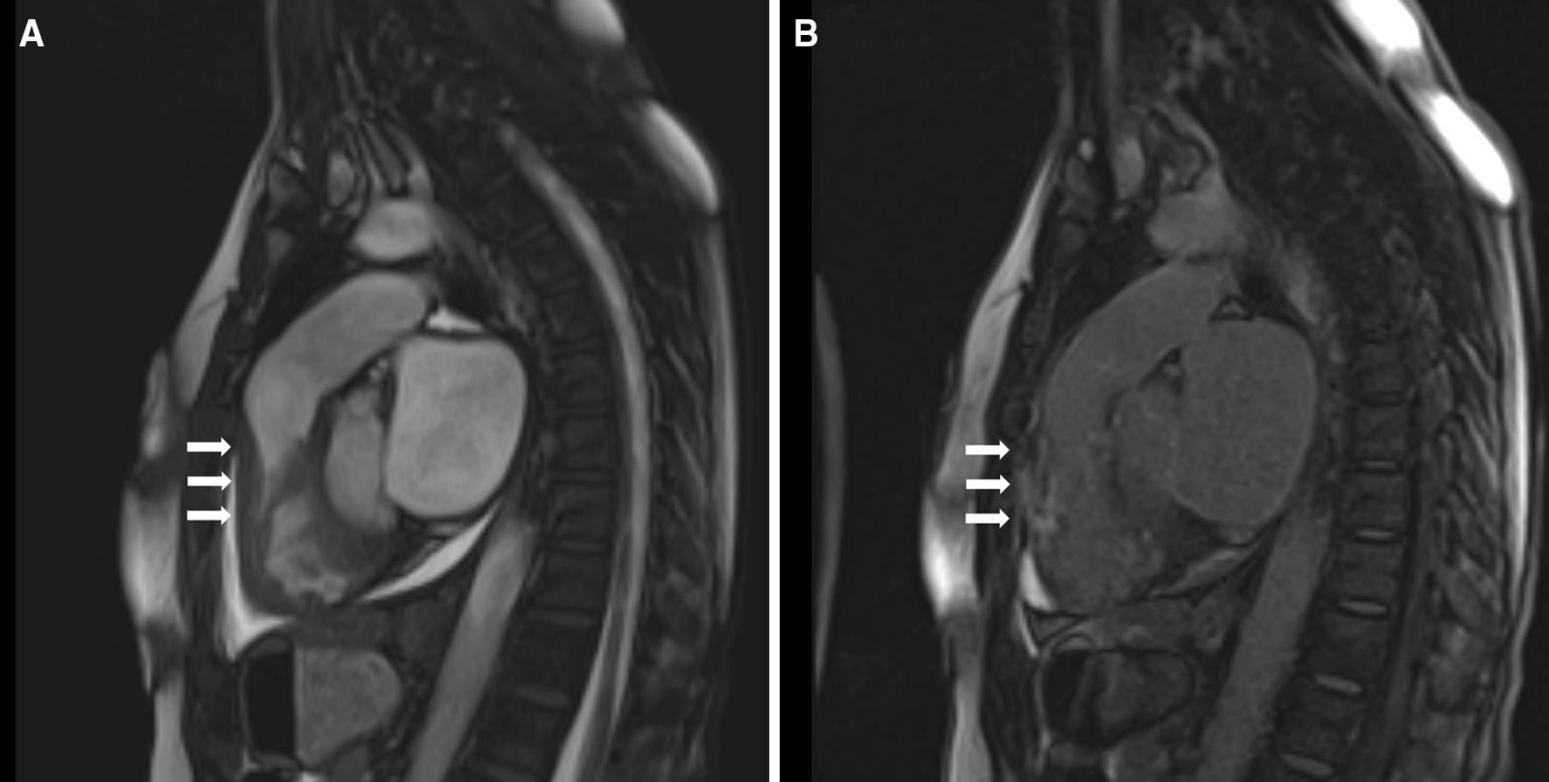
Representative images showing right ventricular hypertrophy (**A**) and late gadolinium enhancement (**B**) in right ventricular outflow tract.

**Figure 3:**
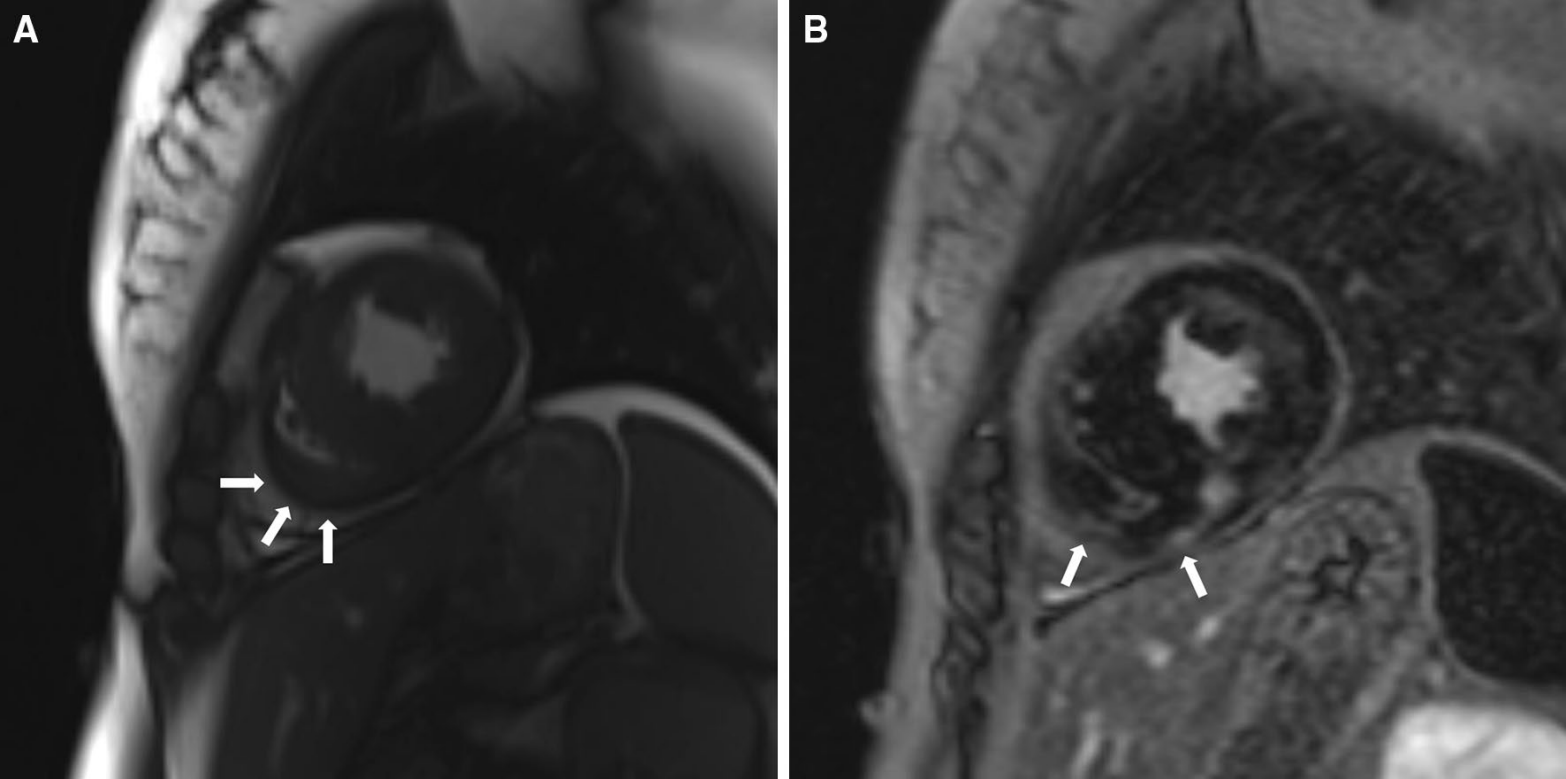
Representative images showing right ventricular hypertrophy (**A**) and late gadolinium enhancement (**B**) in the inferior and anterior wall of the right ventricle.

LGE in the LV was present in 227 (89.7%) patients, and RV LGE was present in 8 (3.1%) patients (p < 0.0001). Representative images showing RV hypertrophy and RV LGE in the apical region (Fig. 1A,B), right ventricular outflow tract (Fig. 2A,B), and inferior and anterior wall of the right ventricle (Fig. 3A,B) are presented.

More than moderate tricuspid regurgitation was present in 15 patients (5.9%). The remaining patients had either moderate (n = 92, 36.4%) or mild/absent (n = 146, 57.7%) tricuspid regurgitation.

Univariate analysis revealed that LVEF, peak LV outflow tract (LVOT) gradient, the presence of LGE in the left ventricle, maximal LV wall thickness, tricuspid regurgitant volume by MRI, NYHA functional class, and probability of SCD at 5 years were positive predictors of RVEF (Table 2). On the other hand, indexed RVEDV and male sex were negative predictors of RVEF (Table 2). Apart from NYHA functional class and probability of SCD at 5 years, all remaining significant parameters in the univariate analysis proved to be significant in the multivariable analysis (Table 2). However, maximal LV wall thickness and the presence of LV LGE were of borderline statistical significance (p = 0.0496 and p = 0.045, respectively).

**Table 2:** Univariate and multivariable analysis of predictors of RVEF.

	Univariate analysis	p-value	Multivariable analysis	p-value
	ß (SE)		ß (SE)	
RVEDV index	− 0.12 (0.03)	0.0001	− 0.14 (0.03)	< 0.0001
LVEF	0.36 (0.06)	< 0.0001	0.31 (0.06)	< 0.0001
Peak LVOT gradient	0.05 (0.01)	< 0.0001	0.03 (0.01)	0.015
LV LGE	6.1 (1.7)	0.0004	3.6 (1.8)	0.0445
Maximal LV wall thickness	0.50 (0.12)	< 0.0001	0.22 (0.1)	0.0496
Sex (for male sex)	− 3.6 (1.0)	0.0006	− 2.3 (0.9)	0.01
Tricuspid regurgitation volume (by MRI)*	0.22 (0.04)	< 0.0001	0.23 (0.03)	< 0.0001
NYHA functional class	1.7 (0.69)	0.01	–	–
Probability of SCD at 5 years	0.41 (0.19)	0.03	–	–
**Model performance**				
R^2^			0.46	
Adjusted R^2^			0.44	

*LVOT* left ventricular outflow tract, *PASP* pulmonary artery systolic pressure, other abbreviations as in Table 1.

*Data available for 210 patients (in the remaining patients, lack of pulmonary flow data precluded measurements).

There were 4 patients (1.6%) with RVEF less than 45%. The only independent predictors of poor RVEF were LVEF (odds ratio = 0.80, 95% confidence interval 0.67–0.95) and tricuspid regurgitation volume (odds ratio = 0.86, 95% confidence interval 0.74–0.99). LVEF ≤ 46% had 100% sensitivity and 98% specificity for the identification of patients with RVEF below 45% (area under the receiver operating characteristic curve = 0.985, 95% confidence interval 0.961–0.996; p < 0.001).

Neither estimated systolic pulmonary artery pressure nor tricuspid regurgitation grade by echocardiography proved to be predictors of RVEF. Additionally, left atrium size did not prove to be a predictor of RVEF (p = 0.91).

In the univariate analysis, indexed LVEDV, male sex, and regurgitant volume were positively correlated with RVEDV (Table 3). RVEF, maximal RV wall thickness, maximal LV wall thickness, the presence of RV LGE, the presence of LV LGE and age were negative predictors of RVEDV. Multivariable analysis revealed that LVEDV and tricuspid regurgitation volume were positive predictors of RVEDV, whereas RVEF, maximal RV wall thickness, LV LGE, and age were negative predictors.

**Table 3:** Univariate and multivariable analysis of predictors of RVEDV.

	Univariate analysis		Multivariable analysis	
	ß (SE)	p-value	ß (SE)	p-value
RVEF	− 0.49 (0.12)	0.0001	− 0.61 (0.10)	< 0.0001
LVEDV index	0.52 (0.04)	< 0.0001	0.41 (0.04)	< 0.0001
Maximum RV wall thickness	− 1.28 (0.42)	0.002	− 1.2 (0.3)	0.0001
Maximum LV wall thickness	− 0.42 (0.24)	0.077	–	–
RV LGE	− 14.9 (5.9)	0.01	–	–
LV LGE	− 9.4 (3.4)	0.006	− 9.8 (3.0)	0.001
Age	− 0.28 (0.07)	< 0.0001	− 0.17 (0.05)	0.0008
Sex (for male sex)	6.1 (2.1)	0.004	–	–
Tricuspid regurgitation volume (by MRI)*	0.28 (0.07)	0.0003	0.34 (0.06)	< 0.0001
**Model performance**				
R^2^			0.55	
Adjusted R^2^			0.54	

*LVEDV* left ventricular end-diastolic volume, *LGE* late gadolinium enhancement, other abbreviations as in Table 1.

*Data available for 210 patients (in the remaining patients, lack of pulmonary flow data precluded measurements).

## Discussion

The main finding of our study is that significant ventricular interdependence exists in patients with HCM. RVEF was significantly associated with LVEF, and in addition to tricuspid regurgitation volume by MRI, the only independent predictor of poor RV ventricular performance (RVEF < 45%) was LVEF. LVEF ≤ 46% had 100% sensitivity and 98% specificity in the identification of patients with RVEF below 45%. Interestingly, the presence of LV LGE, maximal LV wall thickness, and peak LVOT gradient were positively correlated with RVEF.

In an experimental model, Damiano et al. showed that LV contraction is of paramount importance for RV-developed pressure and volume outflow^7^. Similar observations have been made by other authors^8^. Several clinical studies in patients with congenital heart disease have demonstrated that the function of the right ventricle depends on the function of the left ventricle^9–15^. However, there are sparse and inconsistent data regarding factors affecting RVEF in patients with HCM. Finocchiaro and colleagues showed that RV dysfunction, defined as an elevated echocardiographic RV myocardial performance index and reduced tricuspid annular plane systolic excursion, was as high as 71%^16^. Additionally, they demonstrated that worse LV function was an independent predictor of RV dysfunction and was of prognostic value in terms of an increased likelihood of death or heart transplantation^16^. The fact that RV dysfunction was associated with adverse outcomes is in line with the study by Hiemstra et al.^17^. Moreover, they demonstrated that the prevalence of RV dysfunction in patients with HCM varied substantially depending on the echocardiographic method of dysfunction assessment and ranged from 5% based on fractional area change to 55% when RV 4-chamber longitudinal strain was implemented^17^. None of the studies, however, assessed the prevalence of poor RV performance measured in MRI imaging in patients with HCM. In our study, there were 1.8% patients with RV dysfunction defined as RVEF below 45%. This may be attributable to the fact that decreased RVEF is a rather late sign of RV dysfunction, and strain analysis is able to detect subclinical ventricular dysfunction before overt systolic dysfunction occurs. A recent study suggested that MRI tissue tracing is able to assess RV deformation in HCM patients and enable the detection of subclinical RV dysfunction prior to RVEF impairment^18^. The usefulness of MRI tissue tracking in the assessment of ventricular function in HCM patients warrants further investigation. Additionally, the contribution of high T2-weighted signal intensity to RV myocardial deformation in HCM patients needs to be investigated^19^. Finally, it should be elucidated whether a novel parameter, namely, the ventricular global function index as determined by MRI, could be useful in detecting subclinical RV function impairment^20^.

Although we did not observe an association between the number of clinical risk factors present in a patient and RV wall thickness, there was a positive correlation between RV wall thickness and the calculated 5-year SCD risk. This association was rather weak (rho = 0.16) but may indicate that patients with RV hypertrophy are at higher risk of SCD. This should be elucidated in future prospective studies with sufficient power to detect such an association.

LV LGE was present in almost 90% of patients, and RV LGE was present in only 3.1% of individuals. The lack of relationship between the presence of RV LGE and RVEF may be due to the small number of patients with LGE located in the right ventricle, in line with a previous report^2^. The presence of LV LGE was associated with higher RVEF and smaller RV cavity. In other words, in patients exhibiting LGE in the left ventricle, we observed a small, hyperkinetic right ventricle. This observation is intriguing. The smaller ventricle must increase the ejection fraction to maintain stroke volume. Additionally, LV LGE was associated with a smaller LV cavity (data not shown); thus, it was indirectly associated with a smaller RV size. Multivariable analysis showed that greater RV wall thickness corresponded to smaller RVEDV.

Tricuspid regurgitant volume measured by MRI was an independent positive predictor of both RVEDV and RVEF, whereas neither estimated systolic pulmonary artery pressure nor tricuspid regurgitation grade by echocardiography proved to be predictors of RV size or function. Tricuspid regurgitation leads to volume overload of the right ventricle and, consequently, to its larger size (higher RVEDV). The reduction in afterload in chronic tricuspid regurgitation actually increases RV stroke volume, although forward flow is reduced. Increased stroke volume translates into higher ejection fraction of the right ventricle. However, it should be underlined that chronic significant tricuspid regurgitation leads ultimately to impairment of RVEF.

The peak LV outflow tract gradient was an independent predictor of higher RVEF. Patients with LV outflow tract obstruction have higher maximum LV thickness^21^ and higher LVEF^22^. In turn, a higher LVEF is associated with a higher RVEF, which explains the higher RVEF in patients with a higher degree of LV outflow tract obstruction.

Finally, we proved that LVEDV indexed for body surface area was positively correlated with RVEDV. The fact that there was a positive association between RVEDV and LVEDV is not surprising since in the absence of severe dilatation of one ventricle, RV size and LV size are correlated and depend on body habitus.

Our study had some limitations. We did not assess the impact of RV function and size on clinical outcomes. This issue warrants further investigation and is addressed in ongoing research. As mentioned above, diminished RVEF is a rather late sign of RV dysfunction. Thus, it may not adequately reflect the true prevalence of poor RV performance in HCM patients. Additionally, only a minority of patients underwent cardiac catheterization (those in whom septal reduction therapy was planned), and we do not have data on filling pressures calculated on the basis of tissue doppler imaging and the E/e’ ratio. Thus, we did not analyse correlations between filling pressures and the RV parameters. Finally, we do not have genetic data for each patient studied; thus, we were unable to provide reasonable analysis between genetic background and RV function and size. This limitation should be addressed in further studies.

In conclusion, RV systolic dysfunction as measured by decreased RVEF is an uncommon feature of HCM and was associated with poor LV systolic function. LV also had a significant impact on RV size, namely, the presence of LV LGE was inversely related to RV size.

## Methods

The study was approved by the ethics committee of the National Institute of Cardiology, and all patients and healthy volunteers provided written informed consent. The study was performed in accordance with the Declaration of Helsinki.

All consecutive patients with HCM referred for cardiac MRI were included. HCM phenocopies (e.g., patients with Fabry disease) were excluded from the analysis. MRI studies were performed with a 1.5T scanner (Avanto/Avanto^fit^, Siemens, Erlangen, Germany). The detailed MRI protocol was as follows: 2-, 3-, and 4-chamber cine images were acquired with a breath-hold electrocardiogram-triggered balanced steady-state free-precession sequence (typical scan parameters: 25 phases, echo time 1.2 ms, repetition time 33–54 ms, flip angle 64°–79°, slice thickness 8 mm, and gap 2 mm). Subsequently, a stack of short axis cine images was obtained covering both ventricles from the base to the apex. This stack was used for RV and LV size and function calculations: endocardial and epicardial boundaries were delineated in end-diastole and end-systole with the use of dedicated software (QMass 7.6, Medis, Leiden, the Netherlands). On the basis of these data, the following parameters were calculated: RV and LV end-diastolic volumes (RVEDV and LVEDV, respectively), RV and LV end-systolic volumes (RVESV and LVESV, respectively) as well as RV and LV ejection fractions (RVEF and LVEF, respectively) and ventricular masses (RVM and LVM). Each volume or mass parameter was indexed for body surface area and expressed as mL/m^2^ or g/m^2^, respectively. Additionally, each image was inspected for the presence of RV and LV hypertrophy, and the maximal wall thickness of the left and right ventricles was recorded. All patients received gadolinium-based contrast agent (gadobutrol, Bayer Pharma AG, Leverkusen, Germany) intravenously at a standard dose (0.1 mmol/kg). Late gadolinium enhancement (LGE) images were acquired 10 to 15 min after injection of gadobutrol. LGE MRI was obtained using a magnitude- and phase-sensitive inversion recovery-prepared steady-state free precession sequence, with the inversion time adjusted to null the normal myocardium. Each acquisition was performed in short and long axis slices in localization identical to cine images.

All MR images were inspected by a physician with considerable experience in cardiac MRI studies (12 years of experience, Level 3 certified expert). The presence of RV and LV LGE was judged on the basis of a consensus of a cardiologist and a radiologist (both Level 3 experts). Intra- and interobserver variability in cine analysis was high (interclass correlation coefficient of 0.99) and was reported previously^23,24^.

A peak LV outflow tract gradient was measured based on echocardiographic Doppler data. Pulmonary artery systolic pressure was determined by the tricuspid regurgitation jet velocity and the estimated pressure in the right atrium based on inferior vena cava diameter and its collapsibility^25^. RV outflow tract or pulmonic valve obstruction was excluded in all patients. In each patient, tricuspid regurgitation was graded by echocardiography (as absent, mild, moderate, or severe) by integrating indices of severity^26,27^. Additionally, regurgitant volume was calculated by MRI by subtracting the pulmonic forward volume from the RV stroke volume.

Clinical data were obtained from of the hospital databases.

Continuous data are presented as the means ± standard deviation (SD) or medians with interquartile ranges (IQR) and were compared using Student’s T-test or the Mann–Whitney test, as appropriate. For comparisons of continuous variables in patients stratified according to NYHA functional class, one-way analysis of variance was used. Categorical variables are presented as absolute numbers and percentages and were compared using the chi-square test. A linear regression analysis was performed to identify factors associated with RVEF or RVEDV. Stepwise multiple regression was conducted with variables that showed a p-value of 0.1 or less in univariate analysis (candidate predictors). The model fit for multiple regression was assessed with the use of R^2^ (coefficient of determination) and adjusted R^2^ (coefficient of determination adjusted for the number of independent variables in a model). Additionally, logistic regression with its accompanying c-statistics, which is equivalent to the area under the receiver operating characteristic curve, was implemented to determine factors associated with impaired systolic RV performance (RVEF < 45%). The Pearson correlation coefficient (r) or Spearman’s coefficient of rank correlation (rho) were used to determine correlations between continuous variables with normal and non-normal distributions. A two-sided p-value of 0.05 or less was indicative of statistical significance. All statistical analyses were performed with Medcalc software (version 19.1.5, Ostend, Belgium).

## Data Availability

The datasets generated and/or analysed during the current study are available from the corresponding author on reasonable request.
